# Adrenocortical Carcinoma and Inferior Vena Cava Thrombosis: Diagnosis and Perioperative Management

**DOI:** 10.7759/cureus.98379

**Published:** 2025-12-03

**Authors:** Joel Kallarackal, Akriti Jalla, Jennifer Caputo-Seidler, John Dahl

**Affiliations:** 1 Internal Medicine, University of South Florida Morsani College of Medicine, Tampa, USA; 2 Internal Medicine-Pediatrics, University of South Florida Morsani College of Medicine, Tampa, USA

**Keywords:** adrenocortical carcinoma (acc), conn’s syndrome, ivc thrombectomy, mitotane, cushing’s syndrome

## Abstract

Adrenocortical carcinoma (ACC) is an endocrine malignancy with a poor prognosis due to its advanced stage at diagnosis and early metastatic potential. Misclassification is common when tumors arise near the renal-adrenal interface, particularly in cases with vascular invasion. We describe the case of a patient with a large retroperitoneal mass extending into the inferior vena cava whose initial imaging suggested a renal origin, resulting in deferred hormonal evaluation. Postoperative pathology confirmed ACC, and subsequent endocrine testing was consistent with a nonfunctional tumor. This case reinforces the need for improved guidelines and increased education, accounting for the variable presentations of ACC, to enhance diagnostic accuracy and optimize pre- and perioperative planning. As such, in cases of patients presenting with masses in the adrenal-adjacent regions, an endocrine workup of cortisol, androgens/estrogens, and mineralocorticoids should be the standard of care to rule out aggressive tumors such as ACC.

## Introduction

Adrenocortical carcinoma (ACC) is a rare and aggressive malignancy, with an estimated annual incidence of 1-2 cases per 1,000,000 individuals [1,2]. This malignancy demonstrates a bimodal distribution with most cases appearing during childhood (under five years) and middle adulthood (fourth and fifth decades) [3,4]. ACC can arise sporadically or in association with hereditary cancer syndromes, including multiple endocrine neoplasia type 1, Lynch syndrome, and familial adenomatous polyposis [1]. Although a higher prevalence is observed in women, the underlying biological mechanisms for this finding remain unclear [1,3]. Clinical manifestations of ACC are often related to hormonal hypersecretion, most commonly cortisol, leading to Cushing’s syndrome [5-7]. Other endocrine presentations may include virilization or feminization due to aberrant androgen or estrogen production, and hypertension with hypokalemia secondary to aldosterone excess (Conn’s syndrome) [8-11]. Nonfunctional tumors (i.e., tumors without hormonal hypersecretion) may present with nonspecific symptoms attributable to mass effect. In acute or emergent cases with high suspicion of malignancy, endocrine evaluation may be deferred to avoid delays in surgical management.

The diagnostic workup for ACC typically involves a comprehensive hormonal assessment and first-line noncontrast CT of the chest, abdomen, and pelvis [12]. ACC with inferior vena cava (IVC) thrombosis presents a unique diagnostic and management challenge, as the disease often manifests at an advanced stage due to its nonspecific symptoms being masked by more common conditions. Vascular invasion further complicates the diagnosis, as this finding may initially suggest alternative etiologies such as renal cell carcinoma (RCC) or hepatocellular carcinoma. Surgical resection with lymphadenectomy remains the mainstay of treatment and is associated with improved recurrence-free survival [12,13]. Adjuvant mitotane treatment, initiated four to six weeks postoperatively, is recommended to reduce the risk of recurrence and limit tumor spread [12,13]. In cases where the disease is not amenable to surgical resection, chemotherapy may be administered in combination with mitotane [3]. A regimen of etoposide, doxorubicin, cisplatin, and mitotane (EDP-M) is the current standard of care for these cases. ACC involving the IVC is particularly uncommon and often mimics RCC due to overlapping radiographic features. Misclassification can delay endocrine workup and alter perioperative planning. This diagnostic challenge underscores the relevance of the present case, in which extensive IVC involvement initially led to a presumed renal origin.

## Case presentation

**Figure 1:**
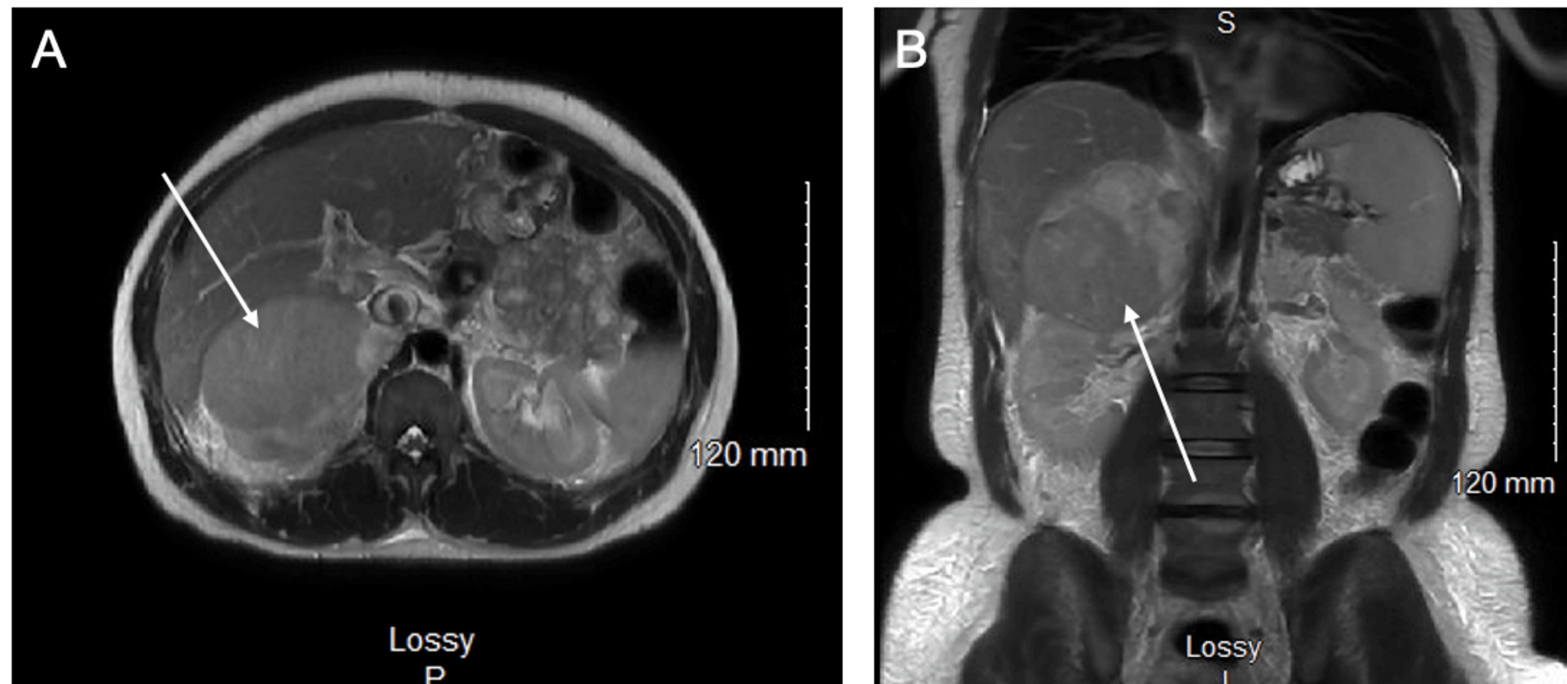
MRI indicating a right adrenal mass. (A) Axial MRI shows an abnormal adrenal mass with mass effect on the posterior right hepatic lobe. (B) Coronal MRI shows the same adrenal mass.

A 49-year-old female with a medical history of sleeve gastrectomy and uterine fibroids presented to the emergency department with a four-week history of bilateral lower extremity edema and generalized abdominal pain. CT of the abdomen and pelvis revealed a 13 × 9 cm right upper pole renal mass with tumor thrombus extending through the right renal vein into the IVC to the level of the inferior cavoatrial junction (Figure 1). Operative management was initiated by urology and surgical oncology for presumed RCC. The patient underwent open right radical nephrectomy and right adrenalectomy with attempted IVC thrombectomy. The thrombectomy was aborted intraoperatively after the thrombus was found to be densely adherent, and further dissection posed a risk of hepatectomy. The operating surgeon noted the tumor in the vena cava to be bulky and hard, contrary to the expected spongy thrombus, prompting concern for ACC. The patient’s postoperative course was uncomplicated.

Final pathology, available on postoperative day 11, confirmed adrenocortical origin. Immunohistochemical staining was positive for steroidogenic factor-1 and synaptophysin, confirming adrenocortical differentiation. The staining also demonstrated strong nuclear positivity for inhibin and vimentin. PAX8 and HMB45 were negative, excluding renal and melanocytic tumors, respectively. Patchy positivity for CK7, CD10, and CA-IX, along with NSE positivity, further supported an adrenocortical phenotype. The Ki-67 proliferative index was 20%, consistent with a moderately high proliferative activity. Histologic evaluation revealed disruption of the reticulin network, a diagnostic feature distinguishing carcinoma from adenoma. The tumor was staged as pT3 pN not assigned (no nodes submitted or found) in accordance with the American Joint Committee on Cancer 8th edition TNM classification [14]. Subsequent postoperative hormonal evaluation yielded results within normal limits (Table 1). The patient was discharged from the hospital with plans for outpatient oncology follow-up for initiation of mitotane therapy and genetic testing.

**Table 1:** Hormonal testing results and reference range. Hormonal testing completed postoperatively measured concentrations of commonly secreted hormones in ACC. Concentrations were found to be within the reference range, indicating low likelihood of a functional tumor in this patient.

Tested hormone	Patient’s value	Reference range
Cortisol (µg/dL)	10.2	2.9–17.3
Testosterone (ng/dL)	13.63	8.70–36.92
Aldosterone (ng/dL)	2	<21

## Discussion

We present a case of ACC in a 49-year-old female whose presenting clinical symptoms of abdominal pain and lower extremity edema were secondary to an extensive IVC thrombus.

As this case illustrates, not all instances of ACC will present with positive hormonal assessments (roughly 40% are nonfunctional), introducing an additional layer of diagnostic complexity. Although radiologic imaging of a large adrenal mass may raise suspicion of ACC, findings are often nonspecific and can overlap with other malignancies. In this patient, preoperative imaging suggested the tumor originated from the right kidney, leading the medical team to prioritize RCC as the most likely diagnosis. Consequently, hormonal assessment was not performed preoperatively [15]. This case underscores a correctable diagnostic pitfall. Routine hormonal screening should be incorporated into the preoperative evaluation of such masses, as imaging alone cannot reliably distinguish ACC from other pathologies [16]. In comparable cases of ACC with IVC involvement, nearly half of the patients presented with excess steroid production, emphasizing the need for prompt hormonal testing.

Endocrine testing was pursued postoperatively once ACC was suspected. Because hormonal studies were obtained after tumor resection, the values shown may not fully reflect the patient’s preoperative functional status. Postoperative testing can underestimate hypersecretion, particularly in cortisol- or aldosterone-producing tumors. Although the patient was obese, Cushing’s syndrome was unlikely due to a postoperative day 10 cortisol level of 10.2 µg/dL. Likewise, virilization was also excluded due to a postoperative day 10 testosterone level of 13.63 ng/dL and the absence of clinical findings. Conn’s syndrome was also ruled out as the patient was within the normal range for aldosterone (2 ng/dL) 11 days postoperatively. Current endocrine guidelines recommend comprehensive hormonal evaluation, including cortisol, androgens/estrogens, and mineralocorticoids, for all adrenal and adrenal-adjacent masses regardless of imaging impression, as biochemical activity may significantly alter perioperative management.

Although this tumor was nonfunctional, the case demonstrates the importance of performing preoperative hormonal testing, as the results hold significant implications for perioperative management. For patients with cortisol excess, thromboembolic prophylaxis, antidiabetic medication, and vaccinations are recommended before surgery [17]. For patients with aldosterone excess, spironolactone administration is recommended to achieve adequate blood pressure preoperatively [17].

Surgical resection via open adrenalectomy remains the treatment of choice to achieve remission in patients with ACC [16]. However, complete resection may not be feasible in patients with an advanced stage or extensive vascular invasion, as seen in this case. In similar cases of ACC with IVC involvement, surgical resection of the primary tumor and IVC thrombus was completed in 80% of patients, but was associated with a 10-15% mortality rate [18]. Several published series describe similar cases in which ACC with extensive IVC invasion was initially misinterpreted as RCC, underscoring the potential for diagnostic error when relying solely on radiographic appearance. The decision to pursue complete resection should be individualized, as it is generally contraindicated in patients with severe comorbidity or unresectable metastatic disease. Recent clinical guidelines suggest neoadjuvant therapy of EDP-M with follow-up evaluation after multiple cycles to determine the risk of resection [16]. Nevertheless, published outcomes remain variable, and the efficacy of this treatment continues to be a subject of debate [19,20].

Clinicians should maintain a high index of suspicion for ACC in patients presenting with retroperitoneal masses. The rarity and heterogeneity of this malignancy can contribute to diagnostic delays and misclassification. Given its aggressive nature and poor prognosis, early recognition is crucial, even in the absence of overt hormonal findings. This case reinforces the need for improved guidelines and increased education, accounting for the variable presentations of ACC, to enhance diagnostic accuracy and optimize both preoperative and perioperative management.

## Conclusions

While the hormonal status of patients remains a major factor in suspected cases of ACC, further recommendations are needed for patients without overt evidence of excess hormone production on clinical evaluation. Although imaging studies are valuable for identifying adrenal masses, they may yield nonspecific findings that can lead to misdiagnosis. To avoid diagnostic delay and enact appropriate management in patients presenting with masses in adrenal-adjacent regions, preoperative hormonal testing and CT imaging must be completed. In this case, the absence of preoperative endocrine evaluation contributed to diagnostic uncertainty, demonstrating how reliance on imaging alone can obscure the true tumor origin. In patients with confirmed ACC, a multidisciplinary approach should be implemented to determine the need for neoadjuvant therapy of EDP-M and long-term management.
